# Multi-Point Laser Detection Device for Ground Hazards in Blind Mobility

**DOI:** 10.3390/s26082396

**Published:** 2026-04-14

**Authors:** Issa Berthe, Lucas Bogaert, Liam Jordan, Julien Donnez, Clément Favey, René Farcy

**Affiliations:** Laboratoire Aimé Cotton, Université Paris-Saclay, Centre National de la Recherche Scientifique, 91405 Orsay, France; lucas.bogaert@universite-paris-saclay.fr (L.B.); liam.jordan@universite-paris-saclay.fr (L.J.); julien.donnez@universite-paris-saclay.fr (J.D.); clement.favey@universite-paris-saclay.fr (C.F.); rene.farcy@universite-paris-saclay.fr (R.F.)

**Keywords:** assistive device, blind mobility, electronic travel aid, laser telemetry, visual impairment

## Abstract

This article examines hazardous ground irregularities that remain undetectable by the white cane used by visually impaired individuals. Additionally, the development of a multi-beam laser ranging system is described. Integrated into the cane handle, this system is designed to provide comprehensive ground awareness and sufficient anticipation at a walking speed of 1 m/s. The system employs a near-infrared multi-beam laser sensor with a holographic grating generating four diamond-shaped beams, in conjunction with a high-resolution CMOS sensor. Through optical triangulation and real-time processing, the device estimates the height of obstacles or drop-offs relative to the walking surface. Vibrotactile feedback informs the user of detected hazards, with distinct vibration patterns differentiating between elevation changes and drop-offs. Preliminary trials with blind participants in controlled environments demonstrate that the system is feasible, responsive, energy-efficient, and fully compatible with conventional white cane use.

## 1. Introduction

Autonomous navigation in unfamiliar environments is challenging and can pose significant risks for visually impaired individuals. The white cane remains the most widely used tool for detecting protruding obstacles or ground-level irregularities. Techniques for cane use have been optimized to minimize the risk of falls. The method developed by Hoover in 1944, which remains in practice today, is described as follows: “The hand holding the cane should be centered on the body. The wrist rotates so that the cane tip traces an arc in front of the user, equidistant from the body center on each side, corresponding to shoulder width. The cane is moved rhythmically in synchrony with the feet, such that when the left foot advances, the cane traces an arc to the right, and when the right foot advances, the cane traces an arc to the left.” The length of a white cane is typically adjusted so that, when held vertically, it extends from the ground up to the user’s sternum, ensuring that the cane tip precedes the foot position [1,2].

**Figure 1:**
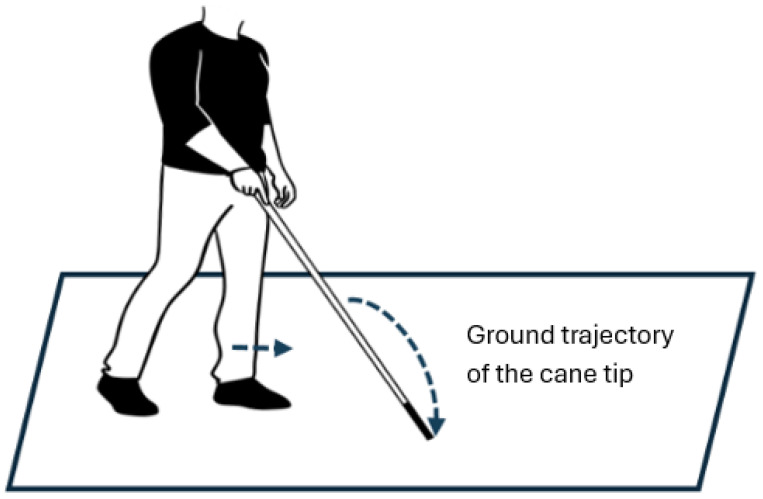
Illustration of the Hoover method.

Figure 1 illustrates the Hoover method, in which advancement of the left foot is synchronized with a rightward sweep of the cane tip to probe the region of the upcoming right foot placement.

Foot placement and cane tip trajectories are simulated using the numerical computation software Octave-9.4.0 and demonstrated via a representative example case study. The simulation parameters involve a user height of 1.70 m and a cane length of 1.30 m inclined at 45°. The shoulder width, assumed to be equal to the width of the cane sweep, is 50 cm, with a step length of 70 cm. The projection of the cane length onto the ground is about 90 cm (130 cm × cos 45°), which corresponds to approximately 1.3 step lengths. Similarly, the height is also 90 cm.

**Figure 2:**
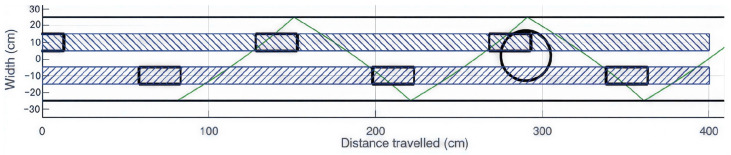
Top-down illustration of the limitations of cane protection when encountering a 30 cm diameter hole in the ideal Hoover configuration.

Figure 2 illustrates the Hoover configuration under ideal conditions, in which the cane trajectory (shown in green) consistently precedes foot placement (black rectangles). The two parallel black lines positioned on either side of the feet are separated by a distance corresponding to the subject’s shoulder width, i.e., 50 cm in the present example. Despite this favorable setup, a hole with a 30 cm diameter (represented by a black circle) may still lead to a fall during the push-off phase, when the toe makes contact with the ground.

**Figure 3:**
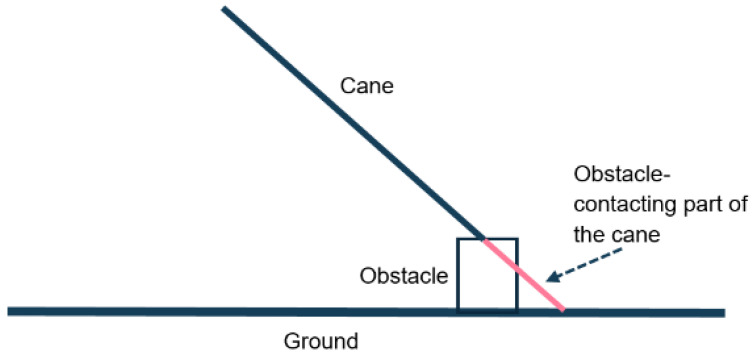
Illustration of the cane section that may contact the protruding obstacle.

In the case of a hole, only the tip of the cane can detect the drop off. For a protruding obstacle, different segments of the cane can potentially detect the obstacle, as illustrated in red in Figure 3. As the obstacle height decreases, the length of the cane segment that may potentially contact it is reduced, making the obstacle more dangerous.

**Figure 4:**
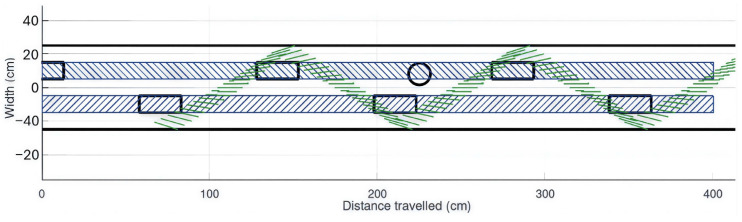
Top-down illustration of the cane’s limited coverage for protruding obstacles at 15 cm height.

In the mathematical simulation shown in Figure 4, the segment of the cane that may come into contact with the protruding object is represented by a green line whose length corresponds to the projection of the segment on the ground. During walking, the foot remains close to the ground, as obstacles only a few centimeters high in the foot’s path may lead to a fall. The black rectangles indicate the position of the feet. The green hatched area in Figure 4 represents the region in which the cane would contact an obstacle 15 cm in height and 13 cm in diameter. Obstacles located outside this region cannot be detected by the cane.

The blue-hatched area represents the trajectory of the feet. The black cylinder of 13 cm diameter denotes an obstacle lying within the foot trajectory that is not detected by the cane.

As illustrated in Figure 4, the detection probability for a salient obstacle (13 cm width, 15 cm height) is low, as the detection zone (green hatched area) fails to fully encompass the foot trajectories (blue-hatched bands). The black rectangles indicate the foot positions during walking, assuming a step length of 70 cm. This analysis demonstrates that the cane provides insufficient coverage for low, protruding obstacles, thereby presenting a potential risk of tripping.

Beyond the issue of non-detection, adequate anticipation is also essential. For the typical user defined above, with a cane length of 130 cm, the cane tip will be approximately 90 cm in front of their feet. Considering an average walking speed of 1 m/s, the user has only 0.9 s to detect the presence of an obstacle and react accordingly. In situations such as descending stairs, this could lead to a dangerous fall if the user is distracted and unable to stop abruptly.

**Figure 5:**
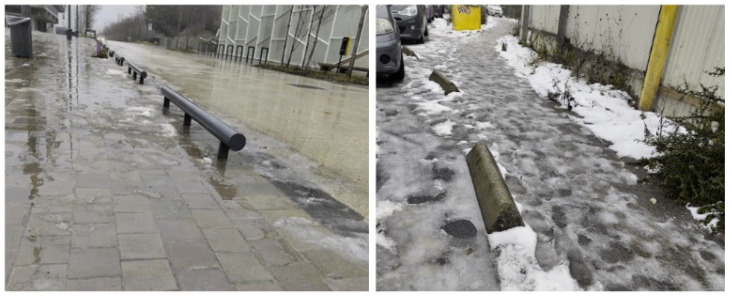
Example of ground-level bollards posing a hazard to white cane users.

Moreover, the ideal Hoover configuration is very rarely achievable in practice. Studies have been conducted with both visually impaired individuals and mobility instructors wearing blindfolds to record foot placement relative to the cane’s trajectory, all using the Hoover technique. They observed, in both groups, a large mismatch between the area previewed by the cane and the actual position of the feet [3,4,5]. These findings help explain the frequent falls experienced by visually impaired individuals due to low street furniture, such as the concrete and steel bollards shown in Figure 5, which are intended to prevent vehicle access in pedestrian areas [6].

To address these limitations, electronic travel aids (ETAs) have been developed to complement or replace the white cane. These systems primarily focus on anticipating obstacles in the horizontal plane and detecting elevated obstacles in addition to the cane. Among the current technologies, ultrasonic systems are the simplest to implement and the most widespread [7,8]. However, in cluttered environments, multiple echoes occur because the ultrasonic waves, which have centimeter-scale wavelengths, reflect strongly off smooth walls, distorting measurements. Rain and high humidity can also produce false positives, as mist or water generates strong echoes. Cane-to-ground vibrations also induce parasitic movements of the ultrasonic receiver’s diaphragm, leading to false positives when attempting to increase sensitivity.

Stereoscopic vision systems enable three-dimensional mapping, but they require high computational power and face challenges in textured or low-light scenes [9].

Regarding active optical telemetry, emission typically occurs in the near-infrared (NIR) range. Several techniques exist. One involves measuring the amount of backscattered light: light pulses of increasing power are emitted, and the pulse that triggers the first reception provides a distance estimate. This technique is sensitive to surface albedo [10], which would be problematic in the context of ground detection as it could generate false positives when transitioning from white road markings to dark asphalt. Another technique is iToF (Indirect Time of Flight), where a modulated light beam is emitted, and the phase shift in the return signal is used to calculate the distance. This technique remains limited to moderate ambient light levels [11]. More recently, integrated dToF (Direct Time of Flight) ranging components based on SPADs (Single-Photon Avalanche Diodes) have been released by STMicroelectronics in single-point and multi-point versions (VL53L series) [12]. However, the narrowest single-point model has an 18° field of view (VL53L4CX), which is too wide for the ground-monitoring application in this study, and the multi-point models generate false positives under high ambient light.

Another approach is laser triangulation, which deduces distance by measuring the displacement of a laser spot’s image on a camera sensor. This has been used to assist the mobility of visually impaired individuals [13]. When used for horizontal plane exploration, it demonstrated immunity to sunlight under real-world operating conditions [14].

Very few studies have focused on complementing ground-level detection by the cane. One device, called the “EyeCane,” provides two beams: one directed toward the ground at 1.5 m and another horizontal beam with a 5 m range [15]. The ground-directed beam replaces the mechanical cane. However, the safety issue related to unexplored ground areas, described at the beginning of the introduction, remains unresolved.

Another approach involves a triangulation system consisting of a laser line projected on the ground and a camera that tracks ground deformation through triangulation [16]. Sweeping motions of the cane are integrated via an inertial measurement unit (IMU), allowing reconstruction of the 3D position of obstacles. This system is limited to indoor spaces because, in compliance with laser safety standards, the emission power distributed along the laser line is insufficient for proper detection by the camera in direct sunlight.

Information feedback is another critical aspect of ETA design. Although auditory feedback is commonly used, it risks masking essential ambient sounds. Vibrotactile interfaces have demonstrated high user acceptance due to their discretion and low cognitive load, particularly when integrated into the cane handle [14].

This article presents an integrated device designed to complement ground exploration via a traditional white cane. The system utilizes laser triangulation via a diamond-shaped four-beam array and is engineered for resilience to high-ambient light, providing enhanced detection of ground topology.

This study will demonstrate that using four beams allows complete protection of the ground against common holes and protruding obstacles through a geometric coverage model. Subsequently, the optoelectronic design and experimental performance of the device are detailed. Finally, the results of preliminary user tests are reported to evaluate the system’s responsiveness and its impact on mobility safety.

## 2. Materials and Methods

### 2.1. Laser Beams Configuration

A configuration of four laser telemetric beams mounted on the cane is proposed to provide anticipation of ground-level obstacles. The initial objective is to verify that the proposed beam distribution fully covers the foot trajectory and significantly improves obstacle detection relative to the use of a traditional cane.

The design utilizes four angularly equidistant beams to facilitate sunlight immunity, a technical requirement justified later in this article.

The selection of the angular spacing, which projects a diamond pattern on the ground, must satisfy several constraints. If the angle is too wide, the lateral beam’s width may prevent the detection of narrow passages, and forward anticipation could become unnecessarily long, potentially hindering mobility. Conversely, if the angle is too narrow, ground coverage is not significantly improved.

**Figure 6:**
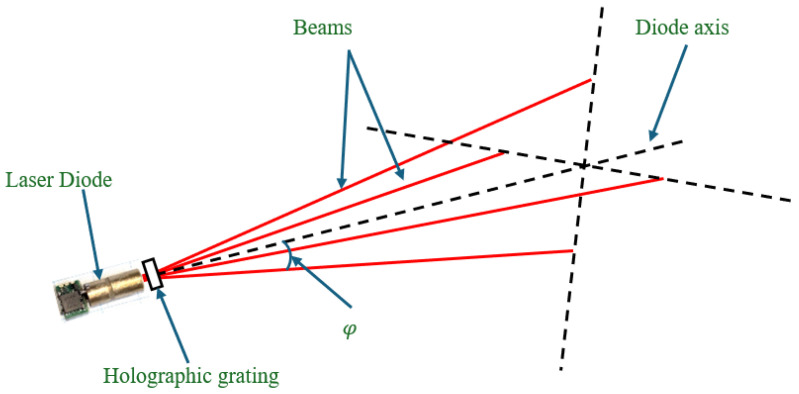
Diagram of the laser beam split into four by the holographic grating.

As shown in Figure 6, four beams are generated from a single-laser diode namely MN850-05FB (Elite Optoelectronics Co., Ltd., Xi’an, China) using a holographic grating MP-002-1-H-A (Holo/Or Ltd., Ness Ziona, Israel), with an angular spacing of 8° relative to the diode’s axis.

**Figure 7:**
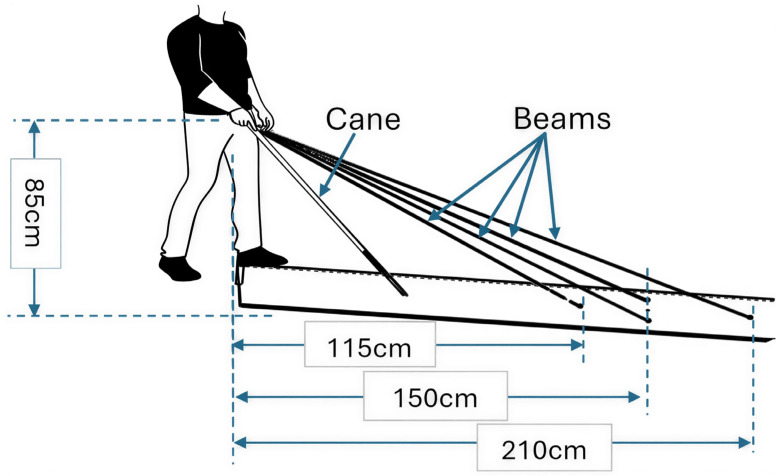
Illustration of the beam distances relative to the cane and the user.

As illustrated in Figure 7, the four beams are positioned ahead of the cane to form a diamond pattern on the ground. The sensor configuration utilizes the following laser spot coordinates relative to the user’s feet: the nearest spot located at 115 cm along the central axis; two lateral spots positioned 150 cm forward and laterally offset by 20 cm on each side of the axis (resulting in a 40 cm spacing between the lateral spots); and the farthest spot along the axis 210 cm in front of the user’s feet.

**Figure 8:**
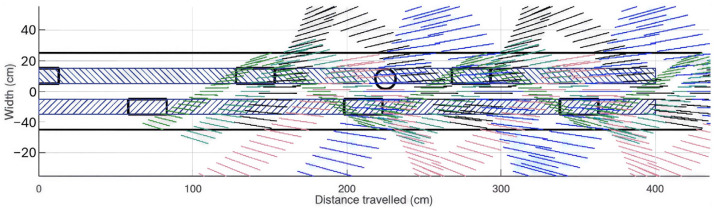
Top-down representation of the coverage provided by the four beams, supplementing the cane for protruding obstacles.

The Octave simulation shows in Figure 8 that this configuration provides complete coverage of a 50 cm wide area along the user’s path, ensuring that obstacles 15 cm in height and 13 cm in diameter are reliably detected.

Figure 8 shows how the coverage provided by the four beams supplements of the cane: cane (green line segments), central beam at 1.15 m (sky blue line segments), right lateral beam at 1.5 m (red line segments), left lateral beam at 1.5 m (black line segments), central beam at 2.1 m (blue line segments), foot trajectory close to the ground during walking (dark blue-hatched bands).

Within the approximately 30 cm wide channel traced by the feet, full coverage can be observed.

**Figure 9:**
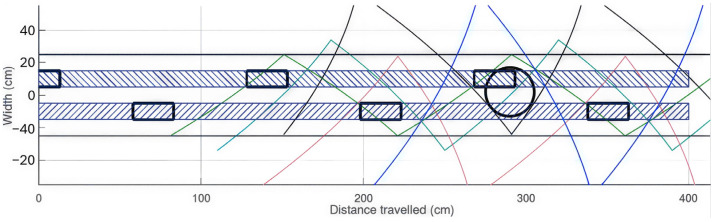
Top-down view of the coverage provided by the four beams, complementing the cane for hole detection.

The protection against holes is evaluated using the situation described in Figure 2, with the addition of the four-beam configuration as illustrated in Figure 9.

It can be observed that a 30 cm diameter hole cannot be positioned so as to intersect the footstep area without being previously detected by one of the laser beams.

With four beams, foot protection remains effective against holes and protruding obstacles even when the Hoover configuration is not respected, which corresponds to the majority of cases, as discussed in the introduction.

A configuration providing complete coverage using only three beams could not be identified. The current choice of the orientation of the four beams is arbitrary; the advantages and disadvantages of positioning the beams further forward or backward will be discussed during testing.

### 2.2. Ranging Technique: Laser Triangulation

Distance measurement by laser triangulation involves pointing a continuous laser beam at the target. The laser spot on the target is imaged by a camera whose optical axis is offset relative to that of the laser. The position of the laser spot in the camera image allows the distance to be determined.

**Figure 10:**
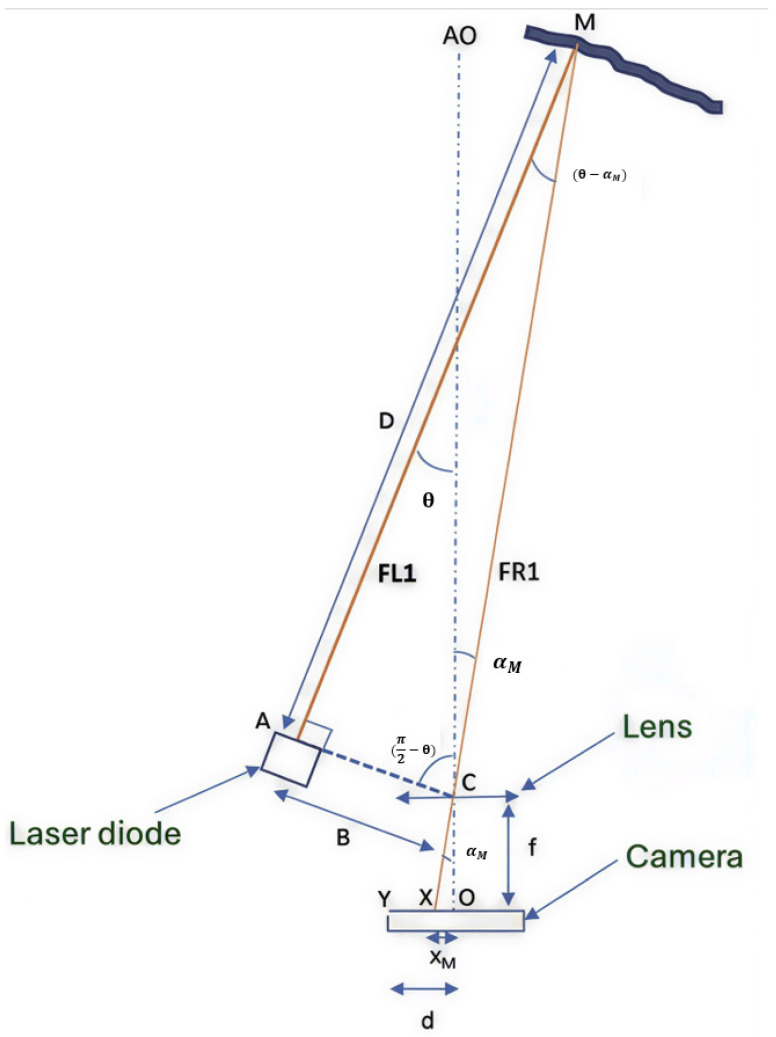
Representation of a laser triangulation system in which the beams are not parallel to the optical axis.

The schematic of a single-beam configuration is presented in Figure 10.

With (*x_M_*, *y_M_*) being the position of the laser spot image on the camera, where the *x*-axis (*OX*) is the intersection of the camera plane with the plane formed by the optical axis and the line (*CA*), with *C* being the center of the camera lens exit surface and *A* the center of the laser exit lens;

*θ* is the inclination of the beam relative to the optical axis;

*D* = *AM* is the distance to the obstacle;

*B* = *CA* is the baseline distance between the receiver and the emitter;

*f* is the focal length of the lens.

Figure 10 shows the schematic diagram for a single beam. The system geometry reveals an initial triangle defined by the optical center of the lens *C*, the optical center of the camera *O*, and the position of the reflected laser spot image *X* on the camera. This triangle is used to determine the field angle *α_M_* at which the impact point *M* is perceived by the camera. This angle is related to the image spot position *X* and the focal length *f* by Equation (1):

(1) $$\tan\left(\alpha_{M}\right)=\;\frac{x_{M}}{f}$$

A second triangle is defined by the optical center of the laser source *A*, the optical center of the lens *C*, and the impact point on the obstacle *M*. Within this triangle, the angle θ represents the inclination of the laser beam relative to the optical axis *CA*. The distance from the obstacle *D*, corresponding to segment *AM*, is a function of θ and the previously determined reception angle $\alpha_{M}$, given by Equation (2):

(2) $$D=B\times \cot\left(\theta -\;\alpha_{M}\right)$$

By replacing $\alpha_{M}$ with its expression derived from Equation (1), the distance *D* is explicitly defined as a function of the laser spot position *x_M_* on the CMOS sensor:

(3) $$D=B\times \cot\left(\theta -\arctan\left(\frac{x_{M}}{f}\right)\right).$$

### 2.3. Optical Design and Resolution

The design objective is to achieve a compact device with a volume of a few cubic centimeters and a resolution tailored for this specific application: the detection of curb drops and significant obstacles. A resolution of a centimeter is considered unnecessary, as the user should not have to interrupt walking for minor ground irregularities such as fallen leaves or small bumps on dirt paths.

The resolution requirements are determined by the need to detect curbside unevenness that could cause falls if detected too late by a white cane. A step or curb is typically 17 cm high, but some curbs can be as low as 10 cm, which can pose a fall hazard. Curbs lower than this are for wheelchair access and do not present a fall hazard.

**Figure 11:**
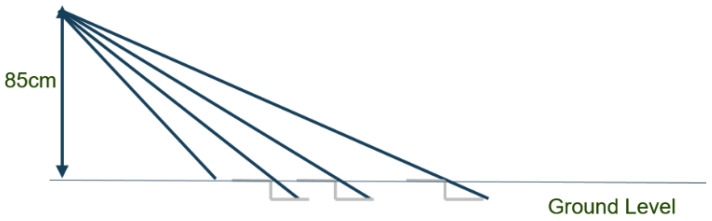
Beam elongation for a 10 cm drop.

Consider a 10 cm high drop (Figure 11) and examine how much each beam lengthens. The beam at 115 cm in front of the feet, inclined at 36°, extends by 10 cm/sin 36° ≈ 17 cm; the beam at 150 cm extends by 20 cm, and the one at 210 cm extends by 27 cm. This provides an order of magnitude for the minimum desired resolution.

The optical characteristics of the system components are as follows.

The CMOS sensor consists of 1920 × 1024 pixels, each 1.4 µm in size. The pixel size represents a compromise between resolution and sensitivity. The active area of the camera sensor measures 2.7 mm × 1.4 mm. The sensor used is the Omnivision OV2722 (OmniVision Technologies, Inc., Santa Clara, CA, USA).

The distance from the laser beam to the camera optical axis, *B*, is 16.2 mm, chosen for sensor compactness.

The angular spacing between the laser beams and the optical axis of the laser is 8°, resulting in a minimum camera field of view of 8 × 8°. Furthermore, increasing the focal length improves spatial resolution. The selected focal length is, therefore, the maximum value that still ensures the minimum field of view of 8°, namely, 3.7 mm.

In addition to field of view considerations, the laser beam divergence determines the size of the projected spot on the camera sensor. Let α be the total divergence of the laser beam; the diameter of the laser spot at distance *D* is α × *D*. Since the distance *D* is significantly larger than the focal length f of the camera, the image of the laser spot is located approximately in the focal plane. The magnification is *f/D*; thus, the spot diameter ϕ is given by α × *f*:

(4) ϕ= α×f.

According to Equation (4), for the optical configuration used in this study, this corresponds to a spot diameter of approximately 4 pixels on the CMOS sensor.

**Figure 12:**
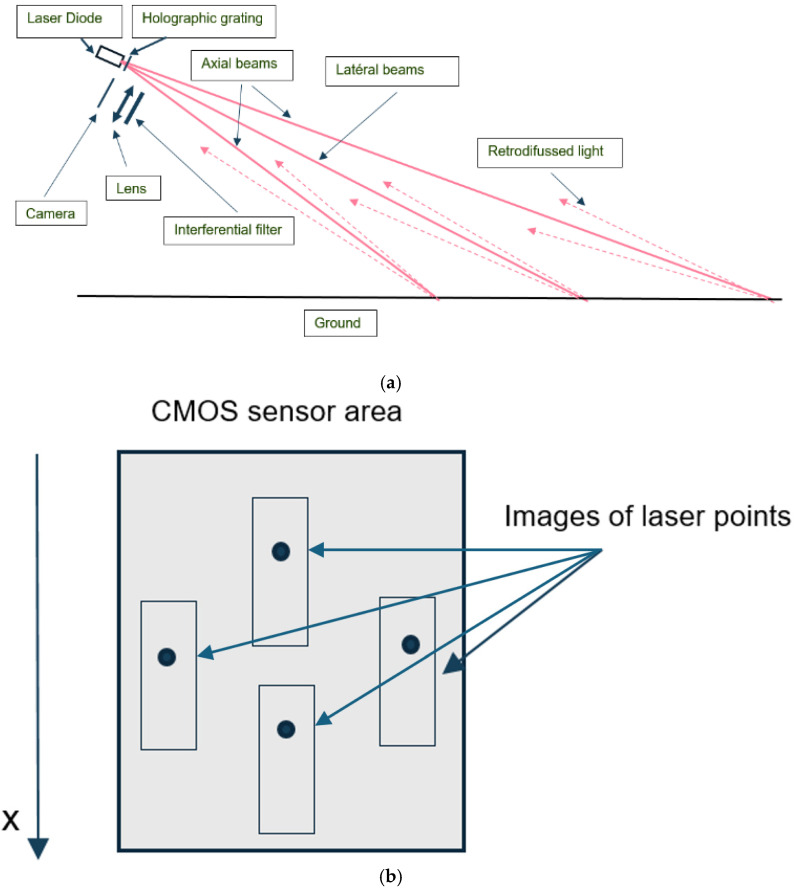
(**a**) Illustration of the system layout. (**b**) Illustration of the sensor area.

Figure 12a,b show a schematic of the optical system component layout and the resulting image on the CMOS sensor.

The four backscattered beams reach the interference filter 85FS10-12.5 (Andover Corporation, Salem, NH, USA), which blocks sunlight, before landing on the camera through the lens M7-3.7mm-Pinhole (Shenzhen Weiweian Technology Co., Ltd., Shenzhen, China).

A decrease in distance results in a downward displacement of the points along the *x*-axis in the camera. The image points remain within the bounding rectangles drawn around them.

The system’s resolution is defined by the derivative of Equation (3) relative to x_M._ Equation (5) resulting from this differentiation provides the analytical sensitivity, representing the change in distance per single-pixel shift:

(5) $$\frac{dD}{dx_{M}}=\frac{B\;\times \;f}{\left(f^{2}\;+\;x_{M}^{2}\right)\;\times \;{sin}^{2}(\theta \;-\;arctan(\frac{x_{M}}{f}))}$$

**Figure 13:**
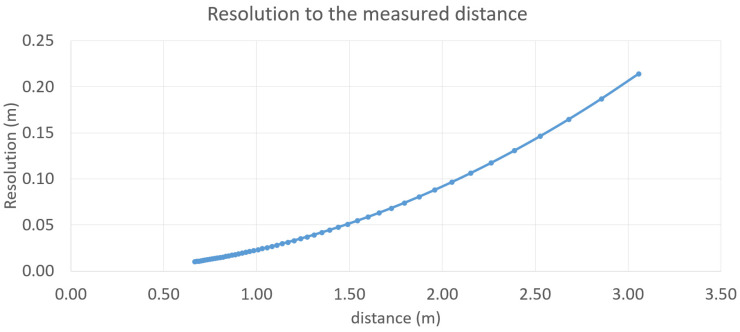
Resolution versus distance for one-pixel shift.

The system’s intrinsic parameters are as follows: baseline *B* = 16.2 mm, focal length *f* = 3.7 mm, and pixel size *p* = 1.4 µm. Equation (5) generates the curve in Figure 13, illustrating the resolution as a function of distance. The curve is plotted for beams with an angle of 8° relative to the camera’s optical axis, as is the case for the four beams in the system of this study.

Table 1 gives the resolution for the distances on a flat floor for each beam, under use conditions.

These values are sufficient for robust detection of a 10 cm drop, considering the values observed in Figure 11. Specifically, regarding the most distant beam, a 10 cm vertical drop from a curb causes a 27 cm extension of the beam (see Figure 11). Consequently, a 13 cm resolution is adequate to detect such curbs.

The laser diode operates at a wavelength of 850 nm, which represents a compromise between the camera’s spectral sensitivity, improved eye safety compared with visible wavelengths, and reduced solar irradiance after atmospheric transmission. Although ambient daylight irradiance is further attenuated at 940 nm, the camera exhibits insufficient sensitivity at this wavelength to ensure reliable detection.

### 2.4. Class I Laser Eye Safety Compliance

The device must be eye-safe in the event of direct beam entry into the pupil, as regulated by the eye-safety standard NF EN 60825-1 [17].

The maximum permissible power for a continuous 850 nm laser beam in Class I is 20 W/m^2^. Given a pupil diameter of 7 mm, the corresponding surface area is approximately 38 mm^2^ at this wavelength. The allowed power for this surface is calculated as 20 W/m^2^ × 38 mm^2^ ≈ 0.77 mW.

Consequently, four beams of 0.75 mW each are generated at an 8° angular spacing from the laser axis, utilizing a holographic grating with 60% efficiency and a 5 mW laser diode.

The angular separation between two beams is 16°. For distances below 25 mm, two beams can enter the eyes; such short distances are not useful for ground control, so a proximity sensor with infrared LEDs, based on the principle described in [10], which is sunlight-resistant, will shut off the laser if an object is very close.

### 2.5. Electronic Architecture

The objective is to develop a system capable of performing the four distance measurements simultaneously while minimizing power consumption.

**Figure 14:**
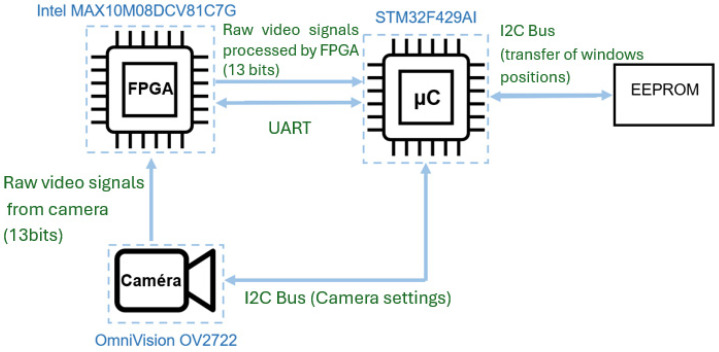
Illustration of the electronic architecture of the sensor.

The architecture, which is highly constrained in terms of power consumption, is based on a low-power 32-bit ARM Cortex-M4 microcontroller (STM32F429AIH6, STMicroelectronics, Geneva, Switzerland) operating at 80 MHz (Figure 14). Since SRAM is the primary contributor to a microcontroller’s power consumption, low-power microcontrollers do not have the capacity to acquire a full image with about 320 kB of SRAM; in fact, 4 MB would be required to store a complete 2-megapixel image at 10-bit resolution.

Moreover, the system only requires access to the image content within four small regions centered around each of the four laser spots. A window of 24 × 112 pixels around each point is sufficient. The window along the *x*-axis, defining the distance range, is 112 pixels wide, while the window width is extended by 24 pixels beyond the image point to ensure a safety margin against mechanical deformation caused by stress and temperature. The four windows together represent 10,752 pixels, i.e., only 0.5% of the total image pixels.

**Figure 15:**
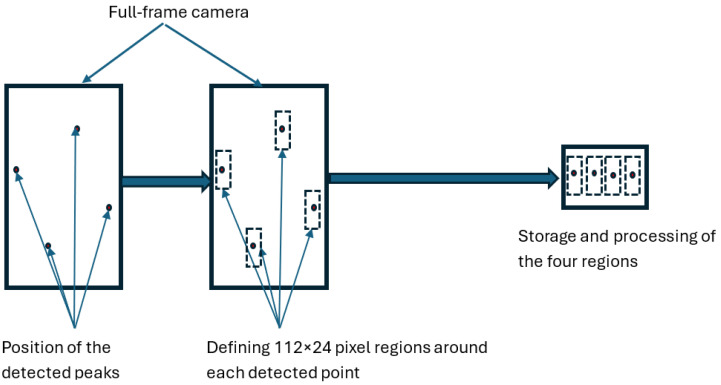
Images of the four beams on the camera with the regions of interest.

Because the microcontroller supports only one acquisition window per image, an FPGA MAX10M08DCV81C7 (Intel Corporation, Santa Clara, CA, USA) is placed between the camera and the microcontroller. The camera data is transmitted to the FPGA, which repackages the signals from each window to present them to the microcontroller as if they were successive small frames. Each full image is thus converted into four small successive images, which are stored in the microcontroller’s memory (Figure 15). This combined microcontroller–FPGA architecture provides a significant reduction in power consumption compared with a microcontroller-based architecture requiring 4 MB of SRAM.

This technique has the advantage of performing the four distance measurements simultaneously. Since the laser emission is continuous, the four points continuously scan the ground during the camera integration time. If the camera integration time is equal to the image refresh period, the beam scans the ground continuously, with no uncovered areas, unlike time-of-flight techniques, where measurements are sampled. If, during the camera integration time, the laser spot, due to the sweeping motion of the cane, encounters two obstacles at different distances, two spots will appear in the camera window, and the distance information for both obstacles is preserved.

The camera clock imposes a minimum readout time of 16 ns per pixel. The camera is read over a total square area of 1000 × 1000 pixels (the maximum area covered by the four points). The image refresh period is 16 ms, with the exposure time adjusted to the same value.

### 2.6. Device Implementation

**Figure 16:**
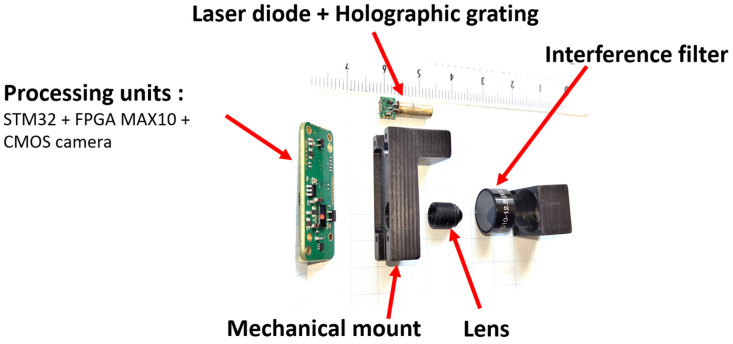
Images of the mechanical assembly of the sensor components.

From a hardware perspective, the sensor unit consists of two electronic boards. The primary board embeds a microcontroller, an FPGA, and a camera for image acquisition and processing. A secondary board is dedicated to the laser diode for power and temperature management. The unit also includes a lens and an interference filter. These components are assembled on a rigid support, as shown in Figure 16, which serves as a mechanical mount to maintain precise alignment between the laser diode, camera, lens, and filter.

Once the hardware is operational, frames from the camera are first processed by the FPGA, which selects four 24 × 112 pixel zones around each beam spot. These four segments are then transmitted to the microcontroller every 16 ms.

These four image segments are processed through an image processing algorithm that converts the raw image into grayscale by applying a moving average over the four neighboring RGGB pixels. Subsequently, a laser spot detection algorithm is executed for each zone. This process involves scanning the area to identify the points of maximum and minimum derivatives; the distance value then corresponds to the pixel located at the midpoint between these two extrema. Because the laser spot image spans at least four pixels with a pseudo-Gaussian profile, a sub-pixel resolution (0.5 pixels) is effectively achievable [18]. Once a spot is identified, its shape is validated over a 10 × 10 neighboring pixel window. The obstacle distance is then directly deduced from the laser spot position.

Finally, the positions of the four laser points are processed by an algorithm to determine if at least one beam has encountered an obstacle. An alert is transmitted to the user via a vibrotactile interface using two distinct vibration patterns: holes are identified by an intermittent 5 Hz vibration, while protruding obstacles trigger a 10 Hz intermittent pattern.

### 2.7. Interference Filter Choice

The central wavelength of an interference filter varies with the angle of incidence of the incoming rays. The four beams are spaced at identical angles so that the backscattered rays from the ground arrive at approximately the same angle on the interference filter, allowing its bandwidth to be minimized.

The device must operate over a temperature range from −30 °C to +45 °C within a limited energy budget that precludes thermal stabilization of the entire system. The wavelength of a laser diode typically increases by about 1 nm for every 3 °C rise in temperature. Using Peltier cooling is not feasible due to power consumption and size constraints. The thermal management approach consists of heating only the laser diode junction to maintain conditions equivalent to an external temperature of 20 °C.

Let λ_C_ be the central wavelength of the interference filter, Δλ its bandwidth and λ_20°C_ the wavelength of the laser diode at 20 °C. The filter is chosen such that:

λ_20 °C_ = λ_C_ − Δλ/2 (6)

Since the beams are inclined by 8°, the wavelength to be considered for λ_C_ corresponds to that of rays incident at 8°, which is approximately 2 nm shorter than for normal incidence.

If an interference filter with a 10 nm bandwidth is selected, at 45 °C, the laser diode wavelength will have increased by approximately 8 nm relative to 20 °C and will still lie within the filter bandwidth. This type of interference filter is common and robust to temperature variations (approximately 0.1 nm increase per 3 °C temperature rise, i.e., about ten times less than that of the laser diode).

### 2.8. Daylight Noise

The following analysis demonstrates that the beams projected toward the ground produce a signal exceeding that of sunlight under maximum illumination conditions.

The maximal total solar irradiance at ground level is approximately 1000 W/m^2^. Based on the irradiance distribution specified in the ASTM G173-03 standard, the spectral irradiance at a wavelength of 850 nm is determined to be as follows [19]:

E_λ_ (ground) = 0.9 Wm^−2^ nm^−1^.

Assuming the use of a 10 nm bandwidth interference filter, the irradiance after the filter becomes:

E_FI_ = 0.9 Wm^−2^ nm^−1^ × 10 nm = 9 Wm^−2^

The diameter of the 850 nm laser spot on the ground for the farthest beam is on the order of 5 mm, corresponding to an illuminated area of approximately 2 × 10^−5^ m^2^. The maximum solar power contained within this area is therefore:

P_FI_ = 9 W m^−2^ × 2 × 10^−5^ m^2^ = 0.18 mW

As previously shown, the power of each individual laser beam is 0.75 mW. The resulting signal-to-noise ratio (SNR) is therefore approximately 4 (0.75/0.18) under the worst-case illumination conditions, ensuring reliable detection even in direct sunlight.

## 3. Results

**Figure 17:**
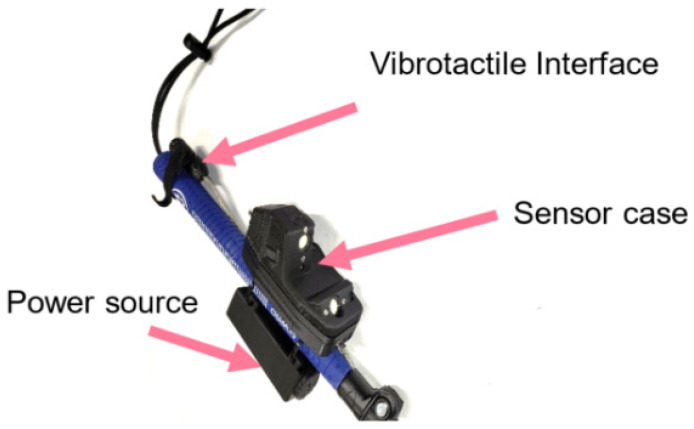
Image of the prototype attached to the handle of a white cane.

The prototype has the following appearance (Figure 17):

### 3.1. Experimental Signal to Noise

The amplitude of the reflected signal depends on the surface reflectance (albedo). The CMOS camera’s sensitivity was experimentally evaluated using the most critical beam, which is the most distant, located at approximately 230 cm with a 23° inclination relative to the ground.

Measurements were conducted using calibrated targets with reflectance of 0.02 (Standard: SRS-02-010 [20]) and 0.5 (Standard: SRS-50-010 [21]), as well as critical urban surfaces such as wet asphalt. The impact of ambient lighting was also assessed under both low and high-intensity conditions.

For each beam, the detection area on the sensor covers 24 × 112 pixels. The camera utilizes an RGGB Bayer filter pattern with 10-bit quantization per pixel. To process the data, the image is converted to grayscale by summing the four neighboring pixels. In indoor conditions, the combined electronic and ambient noise level is approximately 70 units, while the maximum measurable amplitude is 4096 (representing sensor saturation).

**Figure 18:**
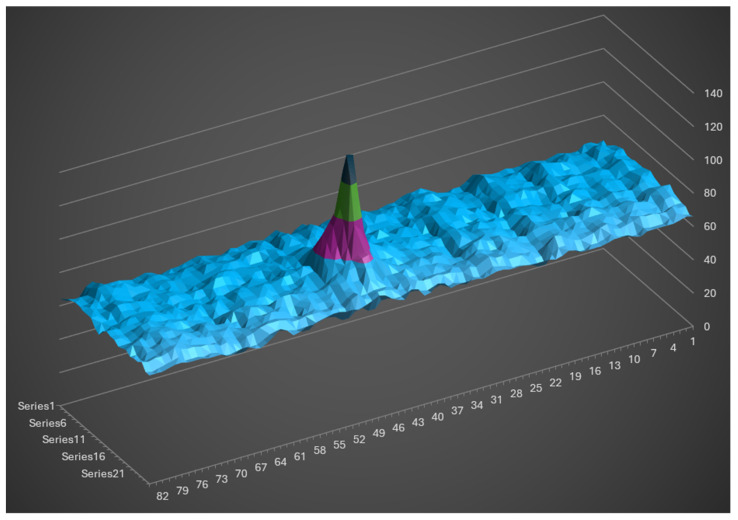
CMOS image on an indoor 0.02 reflectance surface.

Figure 18 displays the laser spot image on a 0.02 reflectance calibrated target indoors. The signal-to-noise ratio (SNR) is greater than 9, which is sufficient for reliable signal detection.

**Figure 19:**
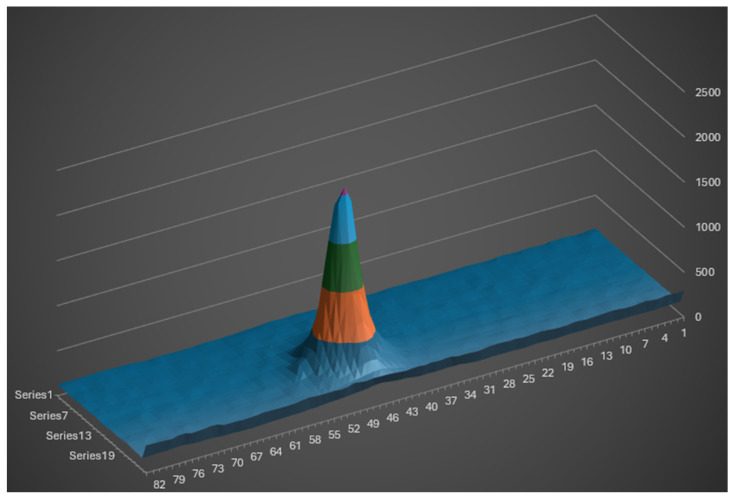
CMOS image on 0.5 reflectance surface indoors.

In contrast, the 0.5 reflectance calibrated target produces a signal amplitude of 2000 against a noise floor of 15 units. Figure 19 illustrates the resulting laser spot image on the camera sensor.

**Figure 20:**
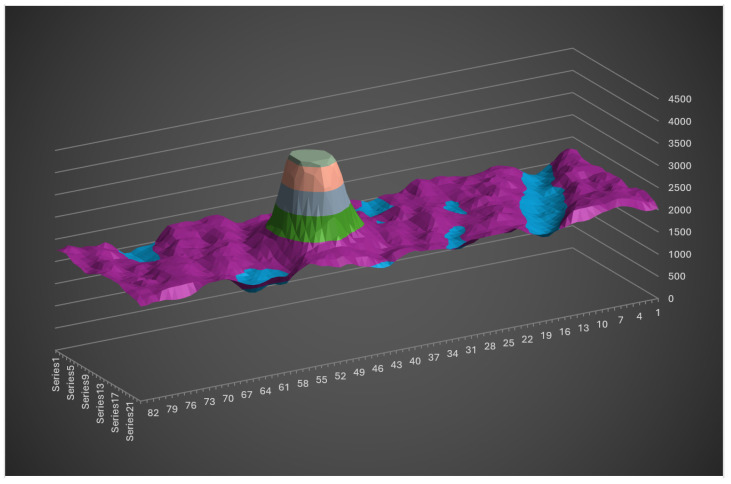
CMOS image of light-colored ground under strong lighting.

Figure 20 shows the image on the CMOS sensor for a light-colored floor under maximal ambient lighting. The signal is 12 bits, and the background is about half of the dynamic range. The image of the laser point is saturated. It remains easily identifiable.

Reducing the CMOS sensor integration time below the frame duration is not recommended to avoid saturation, as it may lead to intermittent ground measurements. Specifically, during the interval between the end of signal integration and the end of the frame, no measurement is acquired, rendering the device temporarily blind.

**Figure 21:**
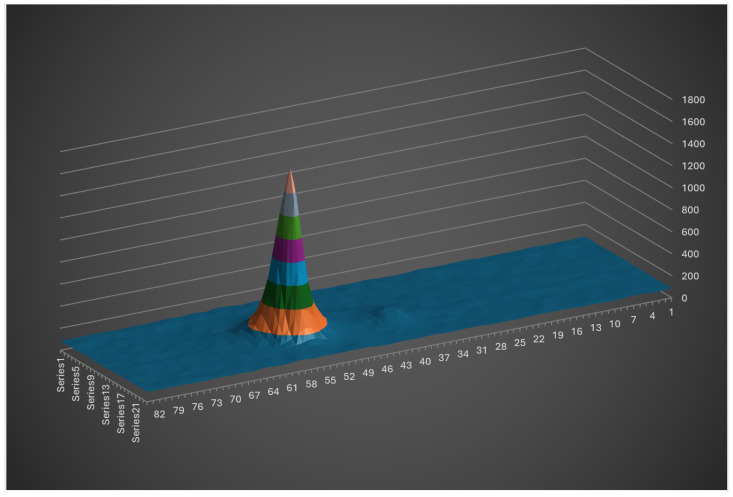
CMOS image of wet dark asphalt under low ambient lighting.

Figure 21 shows the signal on wet dark asphalt under low ambient lighting, corresponding to an overcast sky. Despite low backscattering (high incidence on a dark, reflective surface), the signal amplitude is sufficient for reliable peak detection.

**Figure 22:**
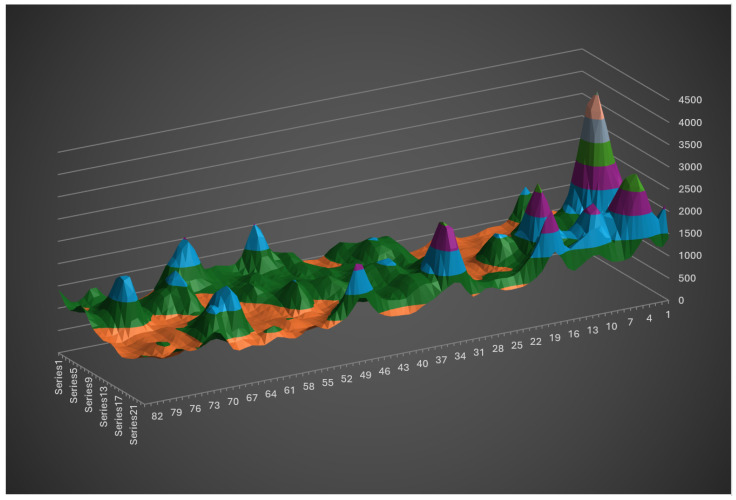
CMOS image of low-angle direct sunlight reflecting off wet asphalt with white stone inclusions.

Figure 22 illustrates the most critical scenario: low-angle direct sunlight reflecting off wet asphalt with white stone inclusions.

The signal intensity remains significantly higher than the spurious peaks, making them easily filterable.

### 3.2. System Calibration

#### 3.2.1. Basic Calibration

Multiple mechanical deformations resulting from physical shocks or thermal expansion can lead to shifts in the laser spot positions on the CMOS sensor. Such misalignments may cause measurement errors, potentially leading to false positives or inaccurate obstacle detection. To mitigate this, a calibration method was developed to reset the reference laser spot positions. The procedure is designed to be performed by the user while equipped with the device. The user maintains the cane in a stationary scanning position, ensuring that their feet, the cane tip, and all four laser beams are positioned on a flat, level floor. The calibration sequence identifies the coordinates of the four highest-intensity pixels, corresponding to the center of each laser spot on the sensor. These coordinates, relative to the full camera frame, are then transmitted to the FPGA. The FPGA utilizes this data to redefine the center of each 24 × 112 pixel processing window, ensuring the tracking algorithm remains recentered on the physical beams despite any minor mechanical shifts.

#### 3.2.2. Complete Calibration

During walking, the height of the user’s hand is inherently unstable due to natural body oscillations. So, it is insufficient to simply define fixed distance intervals around the initial flat-ground calibration values. Furthermore, users typically raise the handle of the cane upward when slowing down, and the cane is often held vertically upon stopping.

It is critical that these variations in the handle height of the cane do not trigger false positives for obstacles or drops. Although an accelerometer could theoretically track the cane’s tilt, raw sensor data is typically too noisy for this application. Implementing the necessary filtering or averaging would introduce latency, compromising the system’s real-time performance.

**Figure 23:**
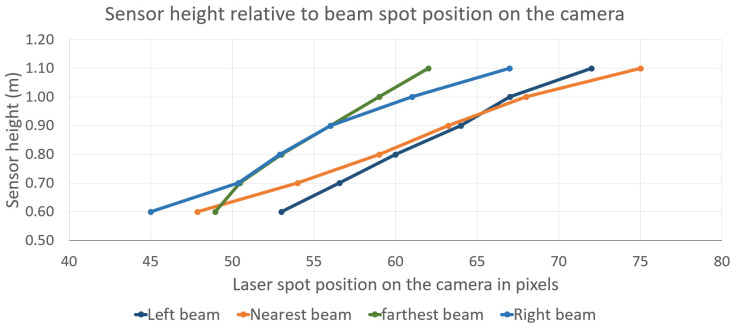
Sensor height on the cane, depending on the position of laser spot on the camera.

To resolve this while maintaining a measurement rate equal to the camera’s frame rate, the system has been characterized by recording the pixel positions of the laser spots across six different heights (Figure 23).

Based on measurements of the four beams across six reference heights, an interpolated curve is derived for each beam to map pixel positions to physical heights. For each frame, the pixel coordinates of the four laser spots are translated into their corresponding height values. If the four heights are identical within a defined tolerance interval, the surface is considered flat; any discrepancies indicate bumps or slopes. This modeling allows the system to distinguish between global height changes (user movement) and local depth variations (obstacles). This straightforward strategy requires minimal computational overhead, ensuring that the system maintains the full video frame rate without introducing latency.

A six-point calibration results in redundant sensitivity, potentially detecting minor objects such as leaves. Furthermore, such a cumbersome procedure is unsuitable for daily users, especially when recalibration is required to fix optical misalignments induced by mechanical shocks, which may happen periodically. Consequently, a simplified two-point calibration protocol was adopted, involving a ‘low’ position (arm extended downward) and a ‘high’ position (cane held vertically). A linear interpolation is then applied between these two reference points. The experimental results presented hereafter were obtained using this streamlined two-point calibration.

### 3.3. Experimental Resolution

The absence of false positives on level ground was initially verified with standard vertical wrist movements, including abrupt transitions of the cane to a vertical position.

To achieve this, a 5 cm tolerance threshold was implemented for the height of the hand holding the cane.

An elevated flat surface (15 cm in height) was subsequently constructed to measure the height interval between the detection thresholds for both positive (bumps) and negative (drops) obstacles. These measurements were conducted at three different cane heights ranging from 80 cm to 110 cm (Table 2).

The system is primarily designed to detect curbs, which typically range from 12 to 15 cm in height. This represents a total detection span of 24 cm, covering −12 cm (drop-offs) and +12 cm (step-ups). The furthest beam is the most critical, yet it maintains a safety factor of approximately two. Conversely, the nearest beam—the most sensitive—must not trigger on negligible obstacles such as leaves. Consequently, a 5 cm sensitivity threshold for this beam represents an acceptable compromise; higher precision is not recommended for this specific method.

## 4. Preliminary Tests by Human and Discussion

### 4.1. Need for an Extra Horizontal Rangefinder

Initial testing of the ground sensor was conducted with the device mounted on the cane without additional ranging systems in an urban environment. This was disorienting for the testers, and several challenges were identified during this phase.

- The detection of both a wall and a low-lying obstacle is initially indistinguishable.
- Effective wall-avoidance strategies for visually impaired users require an anticipation distance of at least 4 m, which significantly exceeds the 2 m range provided by the ground sensor [22].
- When facing a car or truck, the beams pass under the vehicle’s chassis, and forward anticipation may be significantly reduced.

### 4.2. Dual Sensor Tests

For the evaluation, a dual-head sensor configuration was developed, incorporating a horizontal head consisting of a single-laser beam device based on a similar measurement principle.

In fact, large frontal obstacles are typically handled with 4–6 m anticipations using a horizontal rangefinder, and require considerable deviations. In contrast, a ground-level hazard can be avoided with a small deviation and rapid return to the initial trajectory.

It was essential to differentiate vibrations originating from the horizontal rangefinder from those produced by floor-level sensors. To achieve this, a continuous vibration was assigned to the horizontal beam, while intermittent vibrations at 5 Hz and 10 Hz signaled drop-offs and protruding obstacles, respectively. This distinct haptic coding enabled users to instantly identify the nature of the obstacle.

These preliminary tests were not structured as clinical trials and were intended solely to identify the main issues. Four participants were involved in the tests: two visually impaired users of the Tom Pouce III [14] with more than ten years’ experience, and two sighted participants tested under blindfolded conditions.

**Figure 24:**
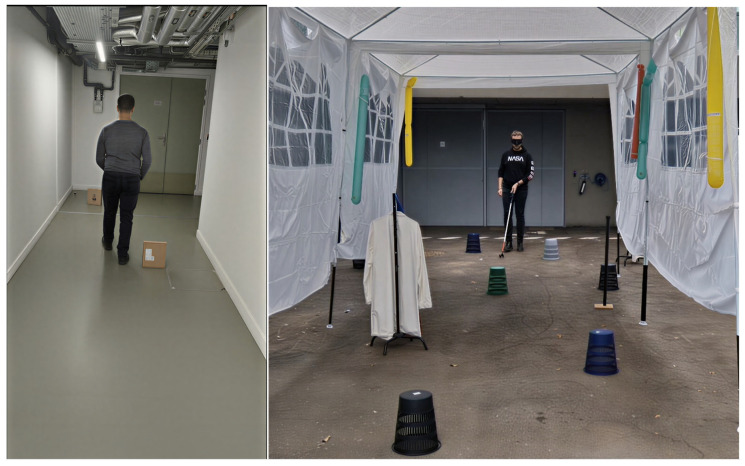
Test corridor with randomly arranged obstacles.

The test consisted of having each participant walk through a corridor of about ten meters with cardboard boxes or small overturned desktop trash bins randomly placed on the floor. The objective was to avoid any mechanical contact with the boxes, either with the body or the white cane. Images of the test environments are shown in Figure 24.

The primary objective of these tests was to observe participants’ behavior during obstacle avoidance. The results indicate that the system is intuitive: naïve users (sighted participants blindfolded) and blind users were able to perform avoidance maneuvers with less than 10% errors in situations where obstacles were spaced more than 2 m apart, that is, when only one obstacle needed to be managed at a time. In this context, the detection range from 1.15 m to 2.10 m appears appropriate and does not impose excessive cognitive load.

In a high-density obstacle environment (more than one obstacle every 2 m), the failure rate increased from less than 5–10% to nearly 30%, although performance remained better for the two visually impaired participants than for the two sighted blindfolded participants.

Some avoidance videos are available at the following link: https://youtu.be/WkJ2EtUl7NY, accessed on 19 February 2026.

In the videos, the two sighted testers are identified by the blindfold.

Regarding common height variations (stair descents, subway or train pits), the user does not need to walk around, but only to stop. A systematic deceleration was observed approximately 2 m before the drop-off.

### 4.3. Discussion

Regarding the calibration algorithm, it was observed that vertical resolution is higher for the near beam than for the far beam. This constraint significantly restricts threshold selection; the shorter beam must filter out ground clutter like leaves, while the further beam must remain sensitive to curb-level drops. Future improvements should aim for a uniform resolution, independent of the specific beam.

Two primary causes were identified for the performance degradation observed with protruding ground obstacles spaced less than 2 m apart.

- While the four-beam configuration ensures a theoretical 100% probability that one beam will encounter the obstacle, the farthest beam is not necessarily the one that encounters the obstacle. As a result, there is an uncertainty of nearly one meter regarding the exact distance to the obstacle. This is not problematic when there is sufficient space to return to the initial trajectory, but if a second nearby obstacle induces a new deviation before the original trajectory is regained, this distance uncertainty prevents precise avoidance.
- A second possible cause is cognitive: the user may focus on the second obstacle before fully managing the first.

Several strategies can be considered to address the decrease in reliability observed among users when navigating environments with numerous closely spaced protruding obstacles at ground level.

- One approach would be to act on the vibrotactile interface by indicating whether the obstacle is detected by a near or far sensing beam.
- Another approach would consist of extending the duration of the vibration over a wider angular range, using gyroscope data, when an obstacle is close, without explicitly conveying distance information, in order to encourage the user to perform a more pronounced avoidance maneuver.

To determine the most appropriate strategy, it is first necessary to systematically identify and characterize real-world situations involving clusters of low-height obstacles.

Regarding drop-off hazards, such as descending stairs or train and subway platform pits, the probability that the largest beam detects the drop first is very high, and the anticipation distance is approximately 2 m compared to the 0.9 m of the cane. At a speed of 1 m/s, the anticipation for downstairs is 2 s instead of 0.9 s with the long cane.

The most notable aspect regarding anticipation is the feeling of comfort and the increase in walking speed in the absence of vibration feedback. Indeed, a white cane may either fail to detect the hazard or detect it with a maximum anticipation of 0.9 s, corresponding to a distance of 90 cm at a walking speed of 1 m/s. At 1 m/s, the cane’s lead time varies from 0 s (for missed hazards) to a maximum of 0.9 s. The proposed device guarantees obstacle detection at a distance between 115 cm and 210 cm, corresponding to an anticipation of 1.15 s to 2.1 s at a walking speed of 1 m/s.

Computational overhead does not pose a challenge for the device, as it utilizes only a low-power microcontroller and a FPGA. The total current draw of the sensor—including the laser diode, camera, microcontroller, and FPGA—is less than 200 mA at 3.3 V.

In terms of environmental diversity, we addressed critical surfaces such as wet dark asphalt and direct sunlight scenarios, which are theoretically the most challenging conditions. While preliminary tests yielded no false positives, even under intense summer sunlight, only long-term trials involving dozens of users across all four seasons will truly identify potential issues in as-yet unforeseen scenarios. In fact, false positives may also arise from ‘clutter’ such as shallow puddles or leaf litter that do not necessitate a user response. The transition from a prototype to widespread, everyday use is typically a multi-year process.

To address potential mechanical or thermal drift over time, we have implemented a user-accessible calibration procedure to restore optimal performance. Ultimately, field data from long-term use will be essential to identify which hardware components require further reinforcement.

## 5. Conclusions

Compared with existing solutions, this system significantly improves the probability of detection for ground hazards thanks to the multi-beam configuration, in comparison to the conventional white cane.

The electronic architecture is capable of 66 measurements per second for each of the four distances, including the filtering process, enabling real-time operation with low power consumption. The system is compact and suitable for integration with a white cane, and it relies on a simple cognitive interface that performs effectively for isolated ground obstacles and height variations.

However, at this stage, a comprehensive field-testing campaign is essential to identify and prioritize improvements necessary to make this device suitable for daily use. Indeed, in such systems, false positives can be highly disruptive. The sources of false positives include: autonomous leaves, potential failures in the solar artifact filtering algorithm, which performs effectively in all conditions, users demonstrating high variability in cane manipulation, attempting to detect minor ground elevation changes that lack practical relevance, increasing the rate of spurious detections, etc.

From another perspective, improving obstacle avoidance in configurations involving closely spaced obstacles will also require a better understanding of real-world scenarios, as well as their occurrence frequency.

The haptic alert structure will be refined through long-term testing in ecologically valid settings.

## Figures and Tables

**Table 1:** Resolution as a function of beam length.

Beam Position and Length	Resolution per Pixel Shift
Near beam, 1.44 m length (1.15 m in front of the feet)	~4.5 cm
Lateral beams, 2.03 m length (1.55 m in front of the feet)	~9 cm
Far beam, 2.48 m length (2.1 m in front of the feet)	~13 cm

**Table 2:** Height interval between positive (bumps) and negative (holes/drops) obstacle alerts.

	Height Interval Between Positive (Bumps) and Negative (Holes/Drops) Obstacle Alerts			
Hand Height	Left Beam (cm)	Nearest Beam (cm)	Farthest Beam (cm)	Right Beam (cm)
80 cm	7.5	5	12.5	8
90 cm	8	5.5	13	8
110 cm	8.5	5	12	7.5

## Data Availability

The original contributions presented in this study are included in the article. Further inquiries can be directed to the corresponding author.
